# AIpollen: An Analytic Website for Pollen Identification Through Convolutional Neural Networks

**DOI:** 10.3390/plants13223118

**Published:** 2024-11-05

**Authors:** Xingchen Yu, Jiawen Zhao, Zhenxiu Xu, Junrong Wei, Qi Wang, Feng Shen, Xiaozeng Yang, Zhonglong Guo

**Affiliations:** 1 Country Co-Innovation Center for Sustainable Forestry in Southern China, College of Life Sciences, Nanjing Forestry University, Nanjing 210037, China; yu567564523@outlook.com (X.Y.); xu123xzx@njfu.edu.cn (Z.X.); wjr781262127@njfu.edu.cn (J.W.); wangqi7553155@gmail.com (Q.W.); 2 State Key Laboratory of Plant Diversity and Specialty Crops, Institute of Botany, Chinese Academy of Sciences, Beijing 100093, China; zhaojiawen0131@gmail.com (J.Z.); yangxz@ibcas.ac.cn (X.Y.); 3 School of Life Sciences, Peking University, Beijing 100871, China; fengshen@pku.edu.cn

**Keywords:** pollen, deep learning, convolutional neural network, ResNet34, AIpollen

## Abstract

With the rapid development of artificial intelligence, deep learning has been widely applied to complex tasks such as computer vision and natural language processing, demonstrating its outstanding performance. This study aims to exploit the high precision and efficiency of deep learning to develop a system for the identification of pollen. To this end, we constructed a dataset across 36 distinct genera. In terms of model selection, we employed a pre-trained ResNet34 network and fine-tuned its architecture to suit our specific task. For the optimization algorithm, we opted for the Adam optimizer and utilized the cross-entropy loss function. Additionally, we implemented ELU activation function, data augmentation, learning rate decay, and early stopping strategies to enhance the training efficiency and generalization capability of the model. After training for 203 epochs, our model achieved an accuracy of 97.01% on the test set and 99.89% on the training set. Further evaluation metrics, such as an F1 score of 95.9%, indicate that the model exhibits good balance and robustness across all categories. To facilitate the use of the model, we develop a user-friendly web interface. Users can upload images of pollen grains through the URL link provided in this article) and immediately receive predicted results of their genus names. Altogether, this study has successfully trained and validated a high-precision pollen grain identification model, providing a powerful tool for the identification of pollen.

## 1. Introduction

Pollen is a critical component in plant reproduction and has significant implications in various scientific fields, including botany, ecology, and allergy research [1,2,3,4,5]. Therefore, understanding and identifying pollen grains can provide valuable insights into plant biodiversity, climate change, and allergen distribution. However, pollen classification presents certain challenges due to the microscopic size and morphological similarity of pollen grains from different species [6]. Accurate identification typically requires specialized expertise and can be time-consuming, highlighting the need for efficient and reliable classification methods. Moreover, manual identification of pollen not only struggles to guarantee accuracy but also incurs significant labor costs and is highly time-consuming. As a result, computer-based pollen recognition is gradually becoming an auxiliary or alternative solution in this field.

With the growing importance of pollen identification, several tools have been developed [7,8,9,10,11,12,13,14,15]. However, many of these tools lack user-friendly interfaces and fail to provide high-accuracy identification (Table S1). This underscores the necessity for more comprehensive and user-friendly solutions in pollen classification. Recent advancements in machine learning and deep learning, particularly Convolutional Neural Networks (CNNs), have revolutionized the field of image recognition [16,17,18]. CNNs have shown exceptional performance in classifying complex image data, making them highly suitable for pollen image identification. This automation removes the need for labor-intensive manual feature extraction and makes the features more representative of the image content, enabling better recognition by the computer. The convolution operation allows CNNs to identify local patterns in images, such as edges or textures, which are then combined in deeper layers to detect more complex structures like shapes or objects. This contrasts with traditional methods that often rely on hand-crafted features, which can be less efficient and less adaptable to diverse image data [19].

Considering the choice of deep learning architectures, significant advancements have been made in image classification, particularly with networks like ResNet34, ConvNeXt, HCGNet, and DenseNet. ResNet34 utilizes a residual network to address the vanishing gradient problem, making it highly efficient for tasks such as pollen classification. ConvNeXt [20] modernizes CNNs by incorporating design improvements inspired by transformers, thereby enhancing feature extraction. HCGNet [21] employs hybrid connectivity and gating mechanisms to balance local and global feature learning, while DenseNet [22] promotes feature reuse through dense connections. ResNet34 is used for its effectiveness and computational efficiency, while the advancements in other networks provide valuable context for future improvements. ResNet34 has been used due to its effectiveness and computational efficiency, and on this basis, we also employed several mainstream methods to enhance the model, including data augmentation and early stopping.

In this study, we developed a robust pollen classification model based on ResNet34, utilizing a substantial dataset of 12,039 preprocessed images that represent 36 genera of pollen. The choice of the ResNet34 architecture proved instrumental in achieving high performance, culminating in an impressive accuracy rate of 97.01%. This accuracy demonstrates the effectiveness of our model in distinguishing between different pollen types, which is essential for various scientific and practical applications. To enhance accessibility and usability for researchers and practitioners in the field, we established the AIpollen website (https://www.aipollen.top (accessed on 15 August 2024)). This platform serves as a user-friendly interface, enabling users to upload pollen images easily and receive rapid classification results. We anticipate that our model and the AIpollen website will serve as valuable resources for researchers of pollen.

In summary, we trained a lightweight ResNet34 model, achieving an average accuracy of 95.97% after multiple training sessions. The model has also been deployed on a website to provide corresponding services to users.

## 2. Materials and Methods

### 2.1. Hardware and Software

The deep learning model employed in AIpollen is developed using the PyTorch (Version 2.5.0+cu118) deep learning framework. The AIpollen website is hosted on a Linux (Version 3.10.0) server powered by Alibaba Cloud technology. Technical support and web application development are implemented using the PHP (Version 7.0.33) language. The back-end servers rely on MySQL (Version 14.14) for efficient data storage and management. The user interfaces of AIpollen are designed with a combination of HTML, CSS, and JavaScript (Version HTML5). Our Python (Version 3.10.11) code primarily utilizes several libraries, including NumPy (Version 1.26.3) and Pandas (Version 2.2.2) for data science tasks, argparse and os for standard library functionality, Pillow (Version 10.2.0) and OpenCV (Version 4.10.0) for image processing, and PyTorch for deep learning applications. Relevant information about these libraries can be found in their respective documentation and forums.

### 2.2. Acquisition and Pre-Processing of Pollen Images

Our image dataset was primarily sourced from pollen-related books and websites, including scanning electron microscopy (SEM) and transmission electron microscopy (TEM) images. The tagging of these images was conducted based on the textual descriptions and guidelines provided in these resources [23,24,25,26]. The dataset includes images from 36 genera of pollen, specifically *Abies*, *Acacia*, *Aster*, *Berberis*, *Camellia*, *Cassia*, *Castanopsis*, *Citrus*, *Clematis*, *Cornus*, *Dendrolobium*, *Eleocarpus*, *Euonymus*, *Faxinus*, *Ilex*, *Indigofera*, *Iris*, *Ligustrum*, *Lonicera*, *Magnolia*, *Michelia*, *Pedicularis*, *Picea*, *Pinus*, *Populus*, *Prunus*, *Quercus*, *Rhododendron*, *Ribes*, *Rosa*, *Salix*, *Symplocos*, *Syringa*, *Tilia*, *Ulmus*, and *Viburnum*. The number of images per class ranges from 30 to 138, with a total dataset comprising 1915 images.

Given the variability in formats and the number of images, it was necessary to pre-process the dataset to ensure uniformity, which is crucial for the performance of neural network models [27]. All images underwent a series of processing procedures to standardize the dataset. Data augmentation has been proven to greatly enhance the performance of deep learning models [28]. Considering that pollen grains under a microscope are typically observed in gray-scale, the first step was to convert all images to gray-scale, reducing the influence of color on model performance. Subsequently, a series of preprocessing steps were applied, including sharpening, contrast enhancement, and random rotation. To account for potential defects in some pollen grains, a portion of the images were randomly masked, covering one-tenth of the area with a mask. After these preprocessing steps, the total number of images increased 12,039, with each class containing between 360 and 400 images. This expansion helped mitigate data imbalance and enhance model robustness. Furthermore, the entire dataset was then divided into training, test, and validation sets in an 8:1:1 ratio, with the validation set used for 5-fold cross-validation. In addition, a data augmentation pipeline was designed with the following sequence of image transformations before submitting the images to the model. The pipeline includes resize image to (256,256) with bi-linear interpolation, center crop image to size of (224,224), random rotate with angles between 0 and 45, random flip vertically with probability of 50%, random flip horizontally with probability of 50%, normalize the images with the ImageNet’s mean and standard variation.

### 2.3. Loss Function and Optimizer

In multi-class classification tasks in deep learning, the CrossEntropyLoss function is one of the most commonly used loss functions. CrossEntropyLoss operates by taking an n-dimensional input, which is initially processed through the softmax function. The softmax function transforms the input into a probability distribution over the class labels. These probabilities are then converted into log probabilities. Finally, the log probabilities are passed to the negative log-likelihood function, which calculates the loss by comparing the predicted log probabilities with the true class labels [29]. This process effectively penalizes incorrect classifications and encourages the model to improve its accuracy over time.

Specifically, the CrossEntropyLoss function optimizes the model’s prediction capability by maximizing the probability of the correct class while minimizing the probabilities of incorrect classes. This loss function is particularly suitable for multi-class problems as it can handle the probabilistic relationships between multiple classes, ensuring that the model remains sensitive to each category’s predictions. As the model learns from the training dataset, the value of the CrossEntropyLoss decreases, reflecting an enhancement in the model’s classification ability and improving overall performance. Consequently, CrossEntropyLoss not only provides an effective feedback signal for the model but also supports convergence throughout the training process.

(1) $$Loss=-\sum_{i=1}^{N}y_{i}log(p_{i})$$

where y_i_ is the one-hot encoding of true labels, and p_i_ is the predicted probability of labels i.

An optimizer refers to the algorithm employed to adjust the model’s parameters based on the gradients of the loss function concerning those parameters. This process is crucial in training deep learning models, as it directly influences their convergence and overall performance. In the context of ResNet architectures, the Adam optimizer was shown to outperform traditional optimization methods such as Stochastic Gradient Descent (SGD) and Momentum, particularly in terms of speed and stability during training [30].

In our work, we utilize the Adaptive Moment Estimation (Adam) optimizer, which combines the advantages of both the Momentum and RMSProp algorithms. The Momentum technique helps to accelerate the optimization process by considering the past gradients, thereby smoothing out the updates and mitigating oscillations. RMSProp, on the other hand, addresses the diminishing learning rates by adapting the step sizes for each parameter based on the recent average of the squared gradients.

By integrating these two approaches, the Adam optimizer not only enhances the efficiency of the optimization process but also stabilizes it, leading to faster convergence and improved model performance. The Adam optimizer updates parameters using the following equations:

(2) $$m_{t}=\beta_{1}m_{t-1}+(1-\beta_{1})g_{t}$$

(3) $$v_{t}=\beta_{2}v_{t-1}+\left(1-\beta_{2}\right)g_{t}^{2}$$

(4) $$\hat{m_{t}}=\frac{m_{t}}{1-\beta_{1}^{t}}$$

(5) $$\hat{v_{t}}=\frac{v_{t}}{1-\beta_{2}^{t}}$$

(6) $$\theta_{t}=\theta_{t-1}-\alpha \frac{\hat{m_{t}}}{\sqrt{\hat{v_{t}}+\epsilon }}$$

where θ_t_ is the parameter, g_t_ is the gradient at the current time step t, α is the learning rate, β_1_ and β_2_ are the decay rates of the momentum term and the gradient squared moving average, respectively, usually taken as β_1_ = 0.9, β_2_ = 0.99, ϵ is a small constant to prevent division by zero errors, usually taken ϵ = 10^−8^.

### 2.4. Learning Rate Scheduler and Early Stopping

In the realm of deep learning, training complex models fundamentally revolves around tackling a non-convex optimization problem. This process typically employs iterative optimization methods designed to converge toward a suitable solution. However, during training, a common challenge arises: the gradients of the model can become exceedingly small. When gradients approach near-zero values, the resulting updates to the model parameters may be minimal, leading to sluggish convergence or causing the model to stagnate within suboptimal regions of the loss landscape.

To mitigate this issue, we implemented a learning rate scheduler. This scheduler reduces the learning rate by half every 20 epochs, thereby allowing for more nuanced updates [31,32]. Such dynamic adjustments to the step size not only facilitate refined tuning of the model but also enhance its capability to escape local optima. As the model approaches convergence, it typically has already assimilated the primary features associated with each class, allowing it to effectively distinguish between most categories. However, continuing the training process beyond this point may lead to the model learning noise rather than meaningful patterns, a situation known as overfitting.

Overfitting is characterized by a scenario where the model demonstrates exceptional performance on the training set while exhibiting a decline in accuracy on the test set. This discrepancy occurs because the model has essentially memorized the training data instead of generalizing from it. To counteract overfitting, we employed early stopping—a strategy that halts training when the loss on the test set ceases to decrease over a specified duration, in this case, 10 epochs [33]. This proactive measure prevents excessive training, helping the model maintain a balance between fitting the training data well and achieving robust generalization on unseen data. Ultimately, these strategies—learning rate scheduling and early stopping—are integral in refining model performance and enhancing its ability to generalize effectively, thus improving overall predictive accuracy on the test set [34].

### 2.5. Modeling Based on Existing Neural Network

To train an effective classification model, four principal components are essential: the dataset for training, a trainable model capable of classifying different classes, an objective function (loss function) to quantify the model’s effectiveness, and an algorithm (optimizer) to modify the model parameters and optimize the loss function. We utilize the ResNet34 model that does not include parameters pre-trained on ImageNet1K and initialized its parameters using the Xavier method. This approach allows for effective training by maintaining the variance of the weights, which helps prevent issues related to vanishing or exploding gradients during the training process. ResNet34 consists of 34 layers, mainly comprising convolutional layers, pooling layers, residual blocks, and fully connected layers. Its structure is as follows [35]:

- Initial convolutional layer: A 7 × 7 convolution kernel with 64 filters and a stride of 2, followed by a 3 × 3 max pooling layer;
- Residual blocks: The network contains 4 stages, each with multiple residual blocks. As the stages progress, the number of channels increases, doubling the number of convolutional kernels;
- Global average pooling: The final layer converts the feature map into a vector.
- Fully connected layer: The output from global pooling is fed into a fully connected layer with 1000 dimensions.

In our adaptation, we replaced the ReLU activation function with the ELU function and modified the final output layer to 36 dimensions to match the number of classes in our study [36]. The hyperparameters are set as follows. Batch size is set as 64. The initial learning rate is set to 0.01, with a learning rate decay [32], which reduces the learning rate by several factors after a specific epoch, applied by multiplying the current learning rate by 0.5 for subsequent learning rates. Although the total epochs are set as 250, we also applied early stopping, which prevents the model from over-fitting [37]. The training procedure stopped when the epoch reached 203. The training loop breaks if the loss value remains below the minimum value for 20 consecutive epochs, resulting in the training loop stopping at the 203rd epoch.

### 2.6. Memory Consumption and Training Time

We trained the initial ResNet34 model on a machine with 16GB of CPU memory and 6 GB of GPU memory. By setting the num_workers to 4, we significantly reduced the data loading time into the DataLoader. During training, the process consumed approximately 14 GB of CPU memory and 5 GB of GPU memory, while testing required about 9 GB of CPU memory and 3 GB of GPU memory. The total training time was around 600 min, with each epoch taking approximately 3 min. With a batch size of 64, each iteration lasted about 1.182 s. The trained model is deployed on a server that utilizes only CPU resources, and it takes approximately 10 s to recognize a single image.

## 3. Results

### 3.1. Model Development for Pollen Identification

For the development of our pollen identification model, we employed ResNet34 as the pre-trained model. ResNet34, the winner of the 2015 ILSVRC competition, achieved an impressive error rate of just 3.5% on the ImageNet dataset. The success of ResNet34 lies in the introduction of residual connections (1 × 1 convolutions) and layer normalization in deeper neural networks. These innovations effectively mitigate the issues of vanishing and exploding gradients, which are common in deep networks. By allowing gradients to flow through shortcut connections, ResNet34 preserves crucial information during backpropagation, enabling the training of much deeper networks without degradation in performance [38,39].

**Figure 1:**
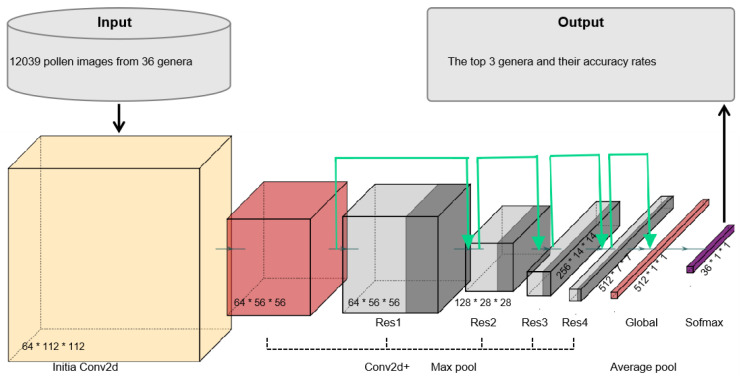
The basic structure of ResNet34, each of which block refers to the shape of output image from this layer.

To tailor ResNet34 for our study, we focused on modifying certain key components to adapt the model to our specific task of pollen classification. First, we replaced the final fully connected layer with a linear layer that features 512 inputs and 36 outputs, which directly correspond to the 36 genera included in our dataset (Figure 1). This modification ensures that the output layer is aligned with the number of classes, making the model capable of distinguishing between the different pollen types in the dataset. Additionally, we replaced the standard ReLU activation function with the Exponential Linear Unit (ELU) function [36], a modification that led to a noticeable increase in classification accuracy from 92% to 97%. The ELU function was shown to offer advantages over ReLU in some contexts, as it helps mitigate the vanishing gradient problem and enables the network to learn more efficiently by introducing a smooth curve for negative inputs.

CNNs have become a dominant approach in many machine learning tasks, especially those related to computer vision. Their ability to automatically learn hierarchical features from raw pixel data makes them particularly well-suited for image classification problems, often outperforming traditional machine learning methods. Unlike conventional algorithms, CNNs can capture spatial hierarchies in data, which are crucial for accurate image recognition tasks, including pollen identification. This strength, combined with the ability to scale with the complexity of the data, has made CNNs indispensable in fields such as medical imaging, object detection, and biological classification.

To further ensure the robustness of our model and to check for any potential biases, we conducted a 5-fold cross-validation on the validation set [40]. Cross-validation is a critical technique for assessing the generalizability of a model and detecting overfitting. Each fold represents a different split of the dataset into training and validation subsets. During this process, the model achieved a validation accuracy of 96.25% on average, with individual fold performances as follows: 95% for the first fold, 95.42% for the second fold, and the same for the third fold, followed by 97.08% for the fourth fold, and a high of 98.33% for the final fold. These results demonstrate that our model generalizes well across different splits of the data and does not display any significant overfitting or underfitting across the validation sets.

In addition to modifying the architecture, we employed several techniques to improve the overall performance of the model. Data augmentation was utilized to artificially expand the dataset by creating modified versions of images through transformations such as rotations, flips, and scaling. This helps the model become more invariant to different visual perspectives and ensures that it can generalize well to new, unseen data. Learning rate scheduling was implemented to gradually reduce the learning rate during training, helping the model converge more effectively. Early stopping was also employed to prevent overfitting by halting training when performance on the validation set no longer improved.

Through these combined techniques, we developed a model that not only achieves high accuracy but also maintains balanced performance across different categories, a crucial factor for practical applications in pollen classification systems.

### 3.2. Model Assessment for Pollen Identification

#### 3.2.1. Key Performance Metrics Analysis

**Figure 2:**
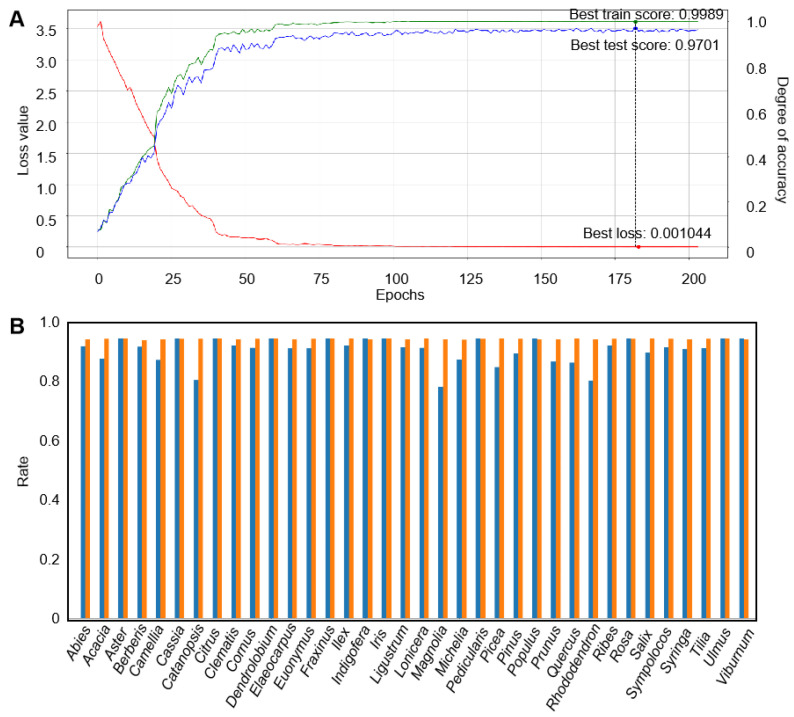
(**A**) The loss value and degree of accuracy over epochs, the black dashed line refers to the model’s highest score on test sets and the lowest loss value. The model may over-fit after this point. (**B**) TPR (True Positive Rate) and TNR (True Negative Rate) for each genus, while the orange bar refers to TNR, the blue means TPR.

*Accuracy.* Accuracy is calculated by taking the ratio of the number of correctly classified categories to the total number of categories, which serves as a fundamental measure of a model’s performance in classification tasks. In our study, after training the model for 203 epochs, we achieved remarkable accuracy rates of 97.01% on the test set and 99.89% on the training sets. These results indicate that the model is highly effective at correctly classifying the majority of pollen images within our dataset (Table 1 and Figure 2A).

The high accuracy rate on the training set suggests that the model has learned the underlying patterns and features associated with the various pollen types, demonstrating its capacity for memorizing and accurately identifying the training data. However, it is equally important to assess the accuracy of the test set, which provides an indication of the model’s ability to generalize to unseen data. The 97.01% accuracy on the test set signifies that the model not only performs well on familiar data but also maintains its efficacy when confronted with new examples.

*MCC.* The Matthews Correlation Coefficient (MCC) is a comprehensive performance metric, especially suitable for imbalanced and multi-class problems [41]. It considers the counts of true positives (TP), false positives (FP), true negatives (TN), and false negatives (FN), providing a more holistic evaluation than accuracy. MCC ranges from −1 to 1: 1 indicating a perfect prediction. The number 0 indicates a random prediction. The number −1 indicates total disagreement between prediction and actual values. Our model achieved an MCC of 0.9573 on the test set, indicating balanced classification performance across all 36 categories without significant bias towards any particular class (Table 1). This demonstrates the high reliability of our model in the pollen classification.

(7) $$MCC=\frac{TP*TN-FP*FN}{\sqrt{\left(TP+FP)(TP+FN)(TN+FP)(TN+FN\right)}}$$

*F1 Score.* In a multi-class classification problem, the F1 score is calculated using the precision and recall for each class, then averaging them. The F1 score is the harmonic mean of precision and recall, measuring the classification performance of the model across different categories [42]. A high F1 score signifies that the model not only accurately detects positive samples but also effectively identifies most of the positive samples. Our model achieved an F1 score of 95.9% on the test set (Table 1). This result indicates that the model has high practical value, capable of minimizing false positives while ensuring the correct identification of the vast majority of positive samples.

(8) $$precision=\frac{TP}{TP+FP}$$

(9) $$recall=\frac{TP}{TP+FN}$$

(10) $$F1\;score=2*\frac{precision*recall}{precision+recall}$$

*ROC AUC.* The area under the receiver operating characteristic curve, Receiver Operating Characteristic—Area Under the Curve (ROC AUC), is a key metric for assessing the discriminative ability of the model [42]. In multi-class classification, the ROC AUC score can be calculated by extending the binary ROC AUC calculation. Since ROC AUC is originally defined for binary classification, multi-class problems require special handling. Here, we use the One-vs.-Rest (OvR) method, where a separate ROC curve is calculated for each class by treating that class as the “positive” class and all other classes as the “negative” class. An AUC value closer to 1 indicates stronger classification capability, allowing better distinction between different categories. Our model achieved a near-perfect ROC AUC of 99.96% on the test set (Table 1). This result demonstrates the extremely high accuracy in classification, effectively reducing the occurrence of misclassifications.

#### 3.2.2. Comparison of the Identification Performance of Pollen Models for Different Genera

**Figure 3:**
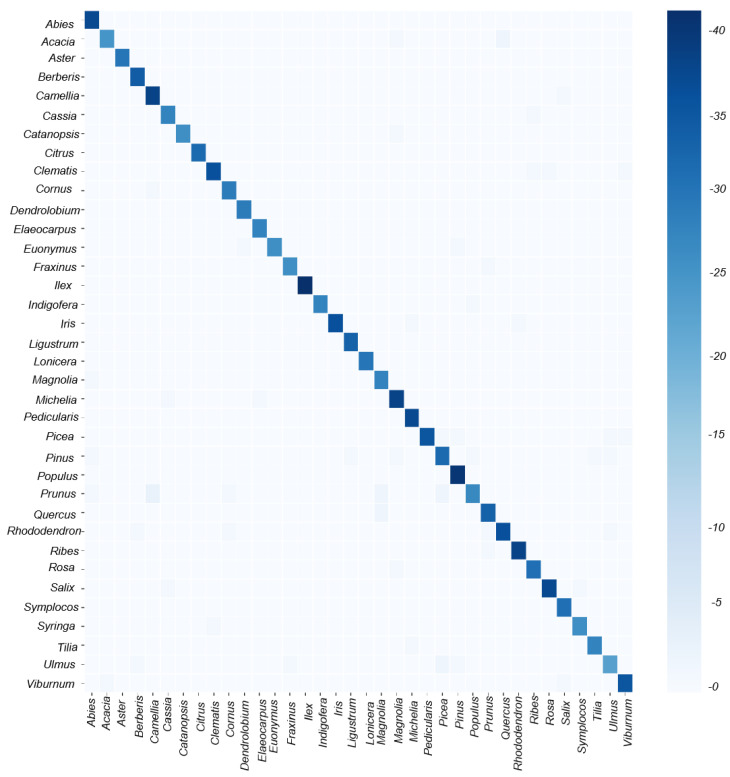
The confusion matrix for final model, the main diagonal indicates the number of correctly classified images out of 40 images for each class.

Although the model exhibits high scores in both F1 score and MCC, indicating robust average performance across all classes, we observe certain preferences for different genera through the TN-TP rates and the averaged confusion matrix (Figure 2B and Figure 3). The best classifications, as observed from the graph, are Aster, Cassia, Citrus, Dendrolobium, Fraxinus, Indigofera, Iris, Pedicularis, Populus, Rosa, Ulmus, and Viburnum, with average accuracies exceeding 95% and perfect TP-TN rates of 100%. In contrast, the worst classifications are Castanopsis, Lonicera, and Rhododendron, with model scores below 87% and TP scores below 82%. Other categories have model scores ranging between 85% and 95%. This variability in phenomenon may be attributed to the varying quality of images and data imbalance. High-quality images and a greater quantity of images for one genus significantly benefit the performance of the model. Additionally, oversampling, which involves replicating samples from the minority class to increase their quantity, is commonly performed using methods like random oversampling or SMOTE (Synthetic Minority Oversampling Technique). SMOTE generates new minority class samples by interpolating between existing ones, effectively increasing diversity and reducing overfitting. Similarly, adjusting class weights by assigning higher weights to the minority class in the cross-entropy loss function ensures that the model pays more attention to the minority class. These techniques could potentially be applied in our future work to improve classification performance on imbalanced datasets.

### 3.3. Overview of Pollen Identification Model Performance

Overall, our model consistently demonstrates high-performance metrics and robust classification capabilities, positioning it as a leading solution for pollen identification. The ability to accurately classify different pollen types with balanced precision and recall across categories indicates that our model is not biased toward any particular class, which is essential for practical applications in real-world scenarios. This robustness is highlighted by impressive metrics, including an accuracy of 0.9701, an MCC of 0.9573, an F1 score of 0.959, and an ROC AUC of 0.9996. Such performance metrics reflect the model’s effectiveness in distinguishing between various pollen types, enhancing its practical utility.

To demonstrate the generalization ability and stability of the model, we reshuffled the dataset and retrained the original model three additional times. This yielded test accuracies of 0.9243, 0.9743, and 0.9701, and training accuracies of 0.9957, 1.0, and 0.9989. Finally, we averaged the accuracies from all four runs, resulting in a final evaluation of 0.9597 for the testing set and 0.9983 for the training set.

Additionally, the analysis of true positive (TP) and true negative (TN) rates reveals that several classes achieve perfect rates of 100%, while a significant portion maintains TP-TN rates around 90%, and only a few categories fall below 85%. This consistency across multiple classes reinforces the model’s capability to handle diverse data inputs effectively. Our findings further emphasize the crucial role that high-quality and balanced datasets play in maintaining consistent performance across all categories.

Imbalanced datasets can often lead to models that are overly sensitive to majority classes, neglecting minority classes and thereby reducing overall accuracy. By ensuring that our dataset is both comprehensive and well-balanced, we mitigate the risks of class bias, ultimately resulting in a model that generalizes better to unseen data.

This approach underscores the need for future studies to focus not only on model architecture but also on careful curation and preprocessing of data to ensure robust performance across all categories. Such an emphasis on data integrity and balance will contribute to more reliable and practical applications in pollen identification systems.

### 3.4. Comparison of ResNet34 with Other Models

Other than ResNet34 with ELU activation function, we trained another mainstream models such as ResNet18 [43], ResNet50 [44], ResNet101 [45], YOLOV8n-cls [46] and YOLOV11n-cls [47] to compare their performance with ResNet34 (Table 2). These models on average perform a little worse than ResNet34, especially for deeper networks such as ResNet101 and YOLOV8n-cls, which may be attributed to their stronger feature-extraction abilities but our less versatile datasets. The model that has reached the most similar scores with ResNet34 is ResNet18, which may be ascribed that its depth resembles the depth of ResNet18. It is worth noting that YOLOV11 is deeper than YOLOV8 but gets a higher score than YOLOV8. In brief, the ResNet34 with an ELU activation function performs better than other models in these 36-classification tasks.

### 3.5. AIpollen Website

**Figure 4:**
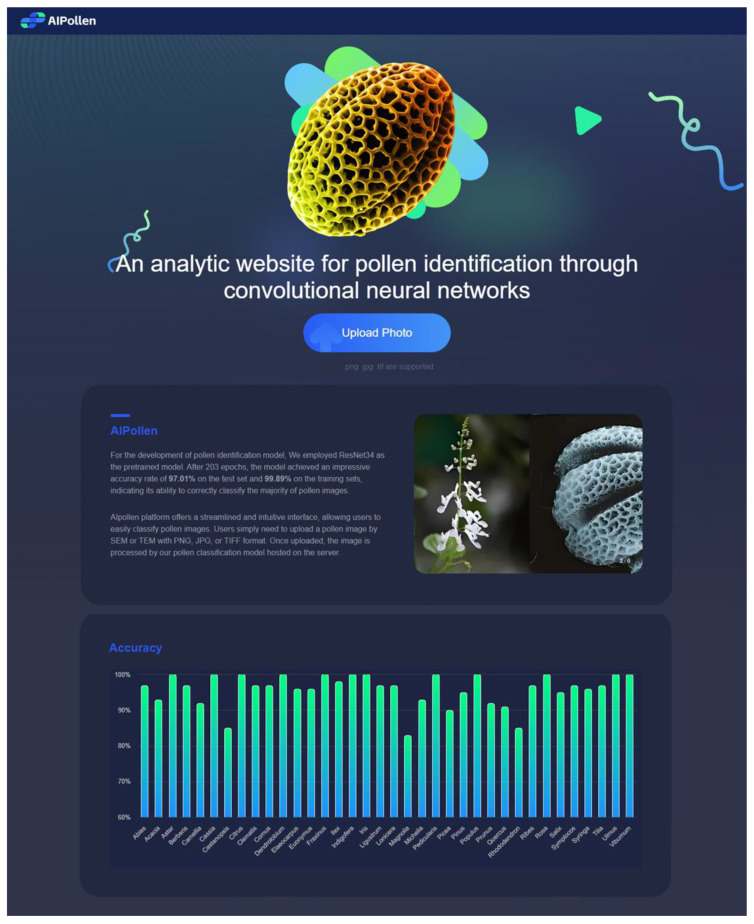
The interface of AIpollen website.

To facilitate the use of our pollen classification model by researchers, we developed the AIpollen website (https://www.aipollen.top (accessed on 15 August 2024)) (Figure 4). The platform offers a streamlined and intuitive interface, allowing users to easily classify pollen images. Users simply need to upload a pollen image in SEM or TEM with a PNG, JPG, or TIFF format (images with formats of SEM and PNG or JPG are the main recommended ones). Once uploaded, the image is processed by our pollen classification model hosted on the server. The website quickly provides the top three classification results along with their respective probabilities, making it an efficient tool for researchers. Additionally, the website features a bar chart displaying the predicted accuracy of different genera. The predicted results and submitted images are stored on the server for future training purposes, enhancing the accuracy of the model over time. The main procedures of the server recognizing the image are described below: The uploaded images will be transferred to the server’s hard disk for storage. Next, a Python script will read these images and carry out a series of operations: the images will be converted to grayscale, resized to 250 × 250 pixels, and then center-cropped to 224 × 224 pixels. Following this, normalization will be applied using the mean and standard deviation derived from the ImageNet1K dataset. Before inputting the images into the model for computation, a new dimension will be inserted into the first dimension (the 0th dimension) of the three-dimensional image tensor, transforming it into a four-dimensional tensor. This step is critical because most deep learning models require input tensors to have a batch dimension, allowing for the simultaneous processing of multiple images. Finally, using our trained model parameters and architecture, the processed images will be identified, enabling accurate classification of the pollen types. This comprehensive pre-processing pipeline ensures that the images are appropriately formatted and normalized, which is essential for achieving optimal performance from the model.

## 4. Discussion

By training other mainstream models, including ResNet18, ResNet50, ResNet101, YOLOV8n-cls and YOLOV11n-cls, we found that ResNet34 with ELU activation function performs better than these more advanced neural networks in this 36-classification tasks (Table 2). Unlike the ReLU function, the ELU function outputs negative values in the negative region, which makes it smoother. From the comparison, we found that CNN with medium depth like ResNet34 performs better than some mainstream models and deeper CNNs in medium classification tasks.

Despite the overall good results, the model still has some limitations in dealing with certain pollen categories due to data imbalances. For genera with a smaller number of images in the dataset, such as *Castanopsis*, *Lonicera*, and *Rhododendron*, the testing accuracy fell below 87%, and the true positive (TP) rate was below 82%. This issue is prevalent in classification tasks with imbalanced datasets, where models tend to perform well on the majority classes but struggle with minority classes. In contrast, for genera with a larger number of images, such as *Aster*, *Cassia*, *Citrus*, and *Dendrolobium*, the model achieved near-perfect results, with an accuracy rate of approximately 95% and a TP-TN rate of 100%.

To address these disparities, future research should focus on implementing cost-sensitive learning. Cost-sensitive learning is a technique that assigns varying misclassification costs to different classes, with higher penalties for misclassifications in minority classes. This approach helps the model to place greater emphasis on correctly identifying minority class samples, thereby reducing bias towards the majority classes [48]. Such adjustments are crucial for improving the model’s performance across all categories and ensuring its robust generalization to different types of pollen. Another approach to tackling class imbalance involves the use of data resampling techniques, such as oversampling the minority class or undersampling the majority class. For instance, SMOTE [49] (Synthetic Minority Oversampling Technique) could be employed to generate synthetic samples for underrepresented classes, thereby increasing their representation in the training data. This would aid the model in learning the characteristics of minority classes more effectively without compromising its performance on majority classes. Furthermore, dataset imbalance can be mitigated through the use of class weighting in the loss function [48]. In this method, higher weights are assigned to minority classes during training, encouraging the model to prioritize the accurate classification of these samples. By implementing such techniques, our aim is to reduce the performance disparity between well-represented and under-represented categories.

Our findings emphasize the importance of having a high-quality and balanced dataset. Imbalanced datasets can lead to biased models, as they tend to be more sensitive to the majority class while neglecting minority classes. This can result in overfitting on the majority class and poor generalization to unseen data. A balanced and comprehensive dataset helps to mitigate these risks by ensuring that the model is exposed to a sufficient number of examples from each class during training [49], thereby enhancing its generalization capabilities.

Currently, our dataset comprises only 36 genera, whereas the actual number of genera in nature is significantly larger. In future research, we intend to expand our dataset by gathering more images of pollen grains from a broader range of genera. This will enable us to train a more generalized model that performs well across a wider spectrum of categories. However, as the dataset size increases, the complexity of the classification task will also rise, making it more challenging for the model to learn to distinguish between different classes. To address this challenge, we may need to explore deeper neural network architectures [50,51], such as DenseNet and ResNeXt, which are capable of extracting more complex features from images. These architectures have proven successful in various computer vision tasks due to their ability to capture hierarchical feature representations, which are essential for differentiating subtle differences in image categories. Deeper networks, combined with regularization techniques like dropout and batch normalization, will help prevent overfitting and improve the model’s capacity to generalize to new, unseen data. Additionally, adopting ensemble learning methods could enhance performance by training multiple models and integrating their predictions to achieve more accurate and robust outcomes. Ensemble methods improve performance by reducing the variance and bias of individual models [52], which is particularly beneficial in imbalanced classification tasks. By averaging the predictions of several models, we can achieve better generalization and minimize the impact of individual model errors.

In summary, while our model currently demonstrates high accuracy rates in the classification of the majority of genera in our dataset, there is scope for improvement, particularly in addressing data imbalance and expanding the dataset to encompass a broader range of pollen types. Future studies will aim to enhance the model’s robustness and generalization capabilities by incorporating techniques such as cost-sensitive learning, deeper network architectures, transfer learning, and ensemble methods. These improvements will be significant in ensuring the model remains effective and maintains high accuracy as it is applied to increasingly diverse and complex datasets in the future.

## 5. Conclusions

In this study, we developed a highly effective pollen classification model, achieving accuracy rates of 97.01% on the testing dataset and 99.89% on the training dataset. Our model not only exceeds the accuracy of most manual identification methods but also outperforms several commonly used neural networks. Additionally, we integrated the model into a user-friendly website, allowing users to easily and accurately recognize pollens.

## Figures and Tables

**Table 1:** Evaluation metrics of the model.

Methods	Classes	Test Accuracy	Train Accuracy	Val Accuracy	MCC	F1 Score	ROC AUC
ResNet34	36	97.01%	99.89%	96.25%	95.73%	95.9%	99.96%

**Table 2:** Evaluation metrics of different models.

Methods	Test Accuracy	Train Accuracy	Val Accuracy	MCC	F1 Score
ResNet34	97.01%	99.89%	96.25%	95.73%	95.9%
ResNet18	98.45%	99.98%	94.33%	94.53%	94.8%
ResNet50	98.53%	99.84%	91.25%	90.95%	91.32%
ResNet101	97.41%	99.87%	92.00%	91.97%	92.35%
YOLOV8n-cls	91.18%	99.18%	91.38%	90.98%	91.45%
YOLOV11n-cls	93.76%	99.17%	93.67%	93.17%	93.89%

## Data Availability

AIpollen is available at http://aipollen.top/ (accessed on 15 August 2024). All source codes are stored on https://github.com/fish-flyer/AIpollen (accessed on 9 October 2024).

## Supplementary Materials

The following supporting information can be downloaded at: https://www.mdpi.com/article/10.3390/plants13223118/s1, Table S1. Comparison of different pollen identification models.
